# A PAX-8-Positive Female Urethral Adenocarcinoma, Intestinal-Type: A Case Report with Diagnostic Challenges and a Review of the Literature

**DOI:** 10.1155/2023/8323821

**Published:** 2023-02-11

**Authors:** Mary M. Torrez, Frances M. Alba, Jain Zhou

**Affiliations:** ^1^Department of Pathology, University of New Mexico, School of Medicine, Albuquerque, NM, USA; ^2^Department of Surgery, Division of Urologic Surgery, University of New Mexico, School of Medicine, Albuquerque, NM, USA

## Abstract

Female urethral adenocarcinoma (FUA) is extremely rare. It is an aggressive malignancy, and clear cell and columnar/mucinous (“intestinal”) represent the two primary histologic subtypes. Diagnosis is often delayed in patients because of their vague symptomatology; hence, they present with an advanced disease and a poor prognosis. The rarity of FUA brings challenges when determining treatment and management, and treatment guidelines for various stages are lacking. We report an intestinal-type FUA that developed from inflammation-related metaplasia in urethral diverticulum with positive paired box 8 (PAX-8) staining. In addition to intestinal-type FUA being extremely rare, this particular entity exhibiting PAX-8 positivity has not been previously described, to the author's best knowledge. The present report highlights the importance of clinical and radiological assessment as well as histomorphologic and immunophenotypic features for an accurate diagnosis of this rare and aggressive malignancy.

## 1. Introduction

Primary urethral carcinoma in females is rare, and it represents 0.02% of cancers in females and <1% of cancers in the female urogenital tract [1]. The most common histologic type is squamous cell carcinoma (SCC) (representing 70% of all cases), followed by transitional cell carcinoma (TCC) (20%) and adenocarcinoma (10%) [1, 2]. Female urethral adenocarcinoma (FUA) is an extremely rare and aggressive malignancy, and the two primary histologic subtypes of FUA are clear cell and columnar/mucinous (“intestinal”) [3]. While the origin remains unclear, previous studies have suggested one possibility to be the periurethral Skene gland (the embryologic homologue to the male prostate gland); others have proposed urethritis glandularis with or without metaplasia [4–7]. To our knowledge, FUA exhibiting colonic adenocarcinoma with paired box 8 (PAX-8) immunoexpression has not been reported to date.

Herein, we report an intestinal-type FUA that developed from inflammation-related metaplasia in urethral diverticulum with positive PAX-8 staining. Because of this distinct immunophenotype, we emphasize the diagnostic accuracy and importance in excluding other sites, including bladder, colorectal, and gynecologic/Mullerian tumors. Additionally, search of PubMed in the English literature of primary FUA was performed, and the accompanying literature review highlights the rarity of intestinal-type FUA.

## 2. Case Presentation

**Figure 1:**
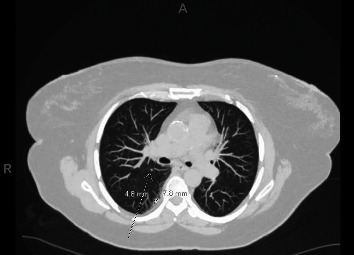
Screening chest computed tomography (CT) scan revealed multiple bilateral pulmonary nodules, measuring up to 7.8 mm.

The patient is a 64-year-old female with a 32-pack-year history of tobacco use who underwent a screening chest computed tomography (CT) scan which revealed multiple bilateral pulmonary nodules, measuring up to 7.8 mm (Figure 1).

**Figure 2:**
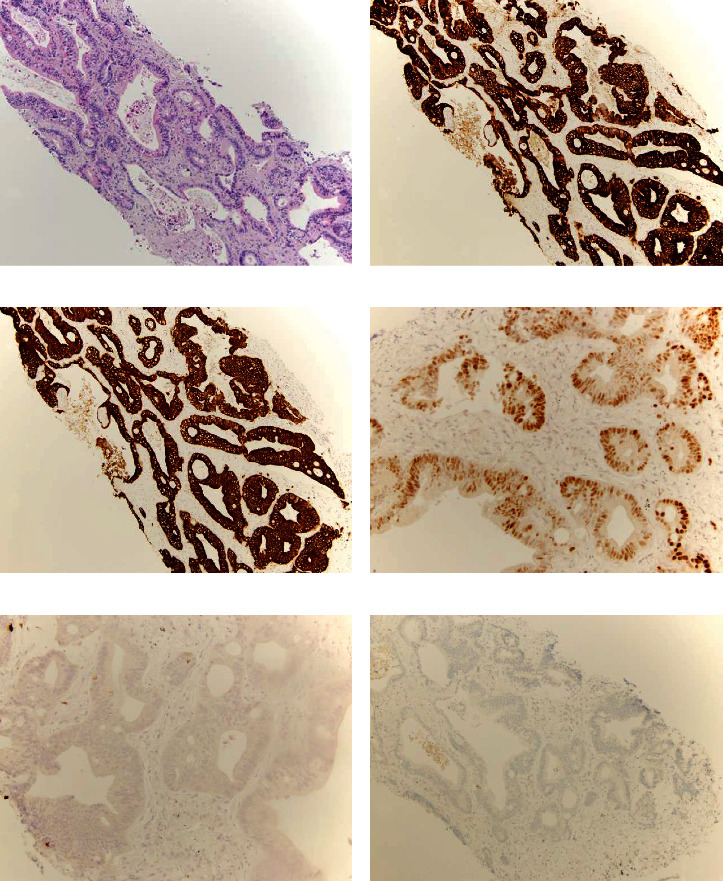
(a) Histologic sections of the needle core biopsy of a right lower lobe nodule demonstrated a proliferation of neoplastic glands with pseudostratified columnar epithelium and intraluminal mucin secretions, consistent with moderately differentiated adenocarcinoma. Immunohistochemistry revealed positivity for cytokeratin 7 (CK7) (b), cytokeratin 20 (CK20) (c), and PAX-8 (d). Transcription termination factor-1 (TTF-1) (e) and estrogen receptor (ER) (f) were negative. Magnifications in (a)–(c) and (f) is 10x and in (d) and (e) is 20x.

Needle core biopsy of a right lower lobe nodule was performed and demonstrated moderately differentiated adenocarcinoma (Figure 2(a)).

**Figure 3:**
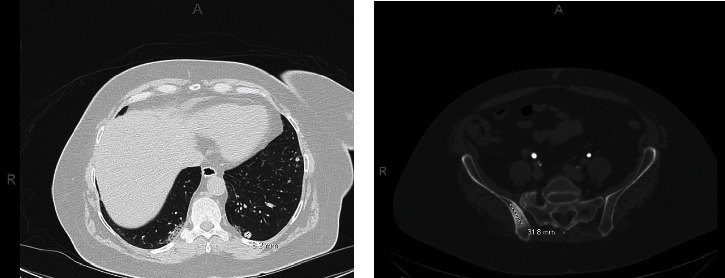
(a) A follow-up CT of the abdomen/pelvis revealed multiple new and enlarging pulmonary nodules in bilateral lungs, and a new sclerotic lesion in the right ilium (b).

Immunohistochemistry (IHC) revealed positivity for cytokeratin 7 (CK7) (Dako/Agilent, Santa Clara, CA, USA), cytokeratin 20 (CK20) (Dako/Agilent, Santa Clara, CA, USA) (Figures 2(b) and 2(c), respectively), special AT-rich sequence-binding protein 2 (SAT-B2) (Sigma, Rocklin, CA, USA), PAX-8 (Abcam, Cambridge, MA, USA) (Figure 2(d)), and negative for TTF-1 (Leica Biosystems, Buffalo Grove, IL, USA) and ER (Ventana Medical Systems, Oro Valley, AZ, USA) (Figures 2(e) and 2(f), respectively); GATA binding protein 3 (GATA3) (Ventana Medical Systems, Oro Valley, AZ, USA) staining appeared cytoplasmic. Because of the immunophenotype, the differential diagnosis included a colorectal primary, and PAX-8 immunoexpression raised the possibility of a gynecologic/Mullerian primary. She had markedly elevated carcinoembryonic antigen (CEA) and cancer antigen 19-9 (CA 19-9) levels. Colonoscopy and extensive imaging were recommended to determine the primary site. Imaging with abdominal and pelvic CT scan showed no evidence of colorectal or gynecologic primary tumors. CT scan and colonoscopy were otherwise normal. A follow-up CT of the abdomen/pelvis revealed multiple new and enlarging solid bilateral pulmonary nodules accompanied by scattered ground glass opacities (Figure 3(a)) and new sclerotic lesions in the right ilium (Figure 3(b)) and vertebral body, concerning for osseous metastasis.

The patient then began having hematuria with intermittent urinary retention. Cystoscopy was performed and showed an irregular/thickened bladder wall. A pelvic exam revealed a firm, fixed urethral mass measuring approximately 4 × 3 cm and invading into the anterior vaginal wall. Cystoscopy revealed that the mass circumferentially involved the urethra and extended up to the bladder neck.

**Figure 4:**
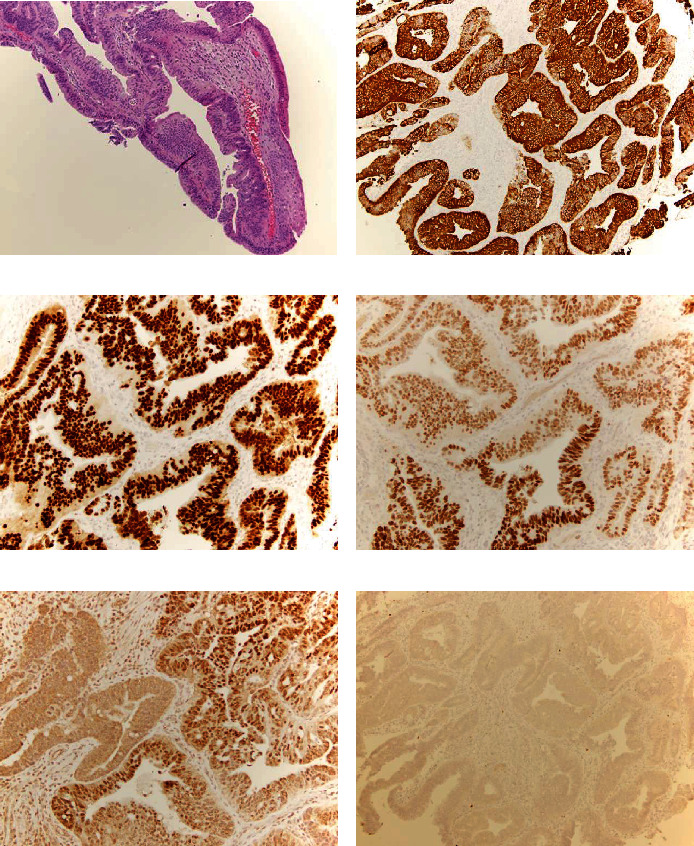
(a) Histologic sections of the urethral biopsy showed intestinal metaplasia of squamous mucosa with transition from a mature to dysplastic phenotype with the adenocarcinoma appearing to originate from the dysplastic intestinal metaplasia. Immunohistochemistry showed positivity for CK20 (b), caudal-type homeobox 2 (CDX2) (c), patchy expression of SAT-B2 (d) and PAX-8 (e), and negative for p16 (f). Magnification in (a), (b), and (f) is 10x and in (c)–(e) is 20x.

There was no disease within the bladder. Urethral and vaginal tumor biopsies were performed. Morphologic examination of the urethral biopsy demonstrated intestinal metaplasia of squamous mucosa with transition from a mature to dysplastic phenotype where the adenocarcinoma originated from (Figure 4(a)).

The vaginal wall biopsy showed the same morphology. The urethral and vaginal wall biopsies showed a similar immunophenotype as the pulmonary nodule biopsy: positive for CK7, CK20 (Figure 4(b)), CDX2 (Ventana Medical Systems, Oro Valley, AZ, USA) (Figure 4(c)), *β*-catenin (BD Sciences, San Jose, CA, USA) (membrane stain), CEA (Biocare Medical, Pacheco, CA, USA), patchy expression of SAT-B2 (Figure 4(d)) and PAX-8 (Figure 4(e)), and negative for TTF-1, p16 (Ventana Medical Systems, Oro Valley, AZ, USA) (Figure 4(f)), ER, vimentin (Dako/Agilent, Santa Clara, CA, USA), and GATA3.

Per the request of the primary oncology team, the case was sent for second pathologic opinion. The consulting service concurred with our diagnosis. This very rare and aggressive subtype warranted further discussion with the tumor board, and a management plan was formulated in consultation with a multidisciplinary team including oncology, surgery, radiology, and pathology. The patient started on the following modified FOLFOX regimen and received it every two weeks: oxaliplatin 85 mg/m^2^ intravenous (IV) day 1, leucocorin 400 mg/m^2^ IV day 1, fluorouracil (FU) 400 mg/m^2^ IV bolus day 1, and FU 2400 mg/m^2^ IV day 1, administered over 46 hours. Cycles 4-6 of oxaliplatin were held due to concern for ocular toxicity and ultimately discontinued upon cycle 13. The patient also received palliative radiotherapy to the lumbar spine and sacral metastases. She suffered from additional complications and side effects secondary to therapy, and the tumor burden progressed. CT and brain magnetic resonance imaging (MRI) demonstrated interval disease progression, and a palliative care consultation was requested.

## 3. Discussion

Female urethral carcinoma is a rare malignancy, and it represents approximately 0.02% of all female cancers and 0.003% of female urogenital malignancies [1, 2]. The reported annual incidence is 1.5 cases per million and increases with age [1]. Among SCC, TCC, and FUA, FUA is exceedingly rare, accounting for approximately 10% of malignant neoplasms involving the female urethra [1].

The female urethral mucosa is lined, from proximal to distal, by transitional, pseudostratified columnar, and squamous epithelia. The composition of the lining epithelium changes from more squamous in young women to predominantly columnar epithelium in the older population. Additionally, paraurethral glands are present in the urethral mucosa and are formed by columnar epithelium or intestinal metaplasia. Although the exact etiology of FUA is unknown, the histogenesis may help explain the pathogenesis. Some have proposed a possible origin from periurethral Skene's glands, while others suggest the epithelium of urethritis glandularis with or without intestinal/glandular metaplasia as a potential source [4–7].

Clinically, signs and symptoms of FUA differ and are often nonspecific. Risk factors are also numerous. The clinical presentation and risk factors are discussed in detail elsewhere [1]. The vague symptomatology can lead to a delay in diagnosis, and patients often present with advanced-stage disease. Even in early-stage disease, this aggressive malignancy is associated with poor outcomes. The rarity of this disease entity creates added challenges when determining treatment options and management, and unfortunately, a general agreement regarding the treatment modality of choice for various stages is lacking [1, 8].

For early-stage disease, partial resection with preservation of the urethra, including local and wide local excisions, is the most common surgical approach, and lymphadenectomies do not affect survival [1]. For advanced-stage disease, treatment has included radical urethrectomy with resection of the paraurethral tissues and anterior vaginal wall. In advanced pathologic stage cancers, there is increased local recurrence following surgery as the only treatment and disease-specific survival is poor [1]. Furthermore, even with extensive and aggressive surgical resections or pelvic exenterations, with or without lymph node dissections, the reported local recurrence rate is 63%. The data for radiotherapy alone is further limited, and when previously used with the intent of cure, local control was not attained [1]. A multimodal treatment approach with surgery, radiation, and systemic therapy (often with multiple chemotherapeutic agents) for patients with advanced disease is commonly practiced. Adjuvant polychemotherapy with gemcitabine, cisplatin, and ifosfamide, or cisplatin, 5-FU, and gemcitabine has been recommended by some [1].

The two primary histologic subtypes of FUA are clear cell type and columnar/mucinous (“intestinal”), with the morphologic features of the former described in detail elsewhere [1]. Columnar/mucinous adenocarcinoma is morphologically similar to well-differentiated colonic or endocervical adenocarcinoma, and the architectural pattern consists of tubular to irregular glands and can also show focal papillary and villous areas. The cytologic features include predominantly atypical columnar cells with eosinophilic to amphophilic cytoplasm, round to oval nuclei with regular contours, and generally small, inconspicuous nucleoli. Mucinous cells are often readily identified and can resemble goblet cells [1, 9]. Rarely, mild to moderate pleomorphism is appreciated and can be associated with large mucin pools and rare malignant glands within [1].

Because of its paucity, immunohistochemical analysis for FUA has not been adequately reported, and characteristic immunophenotypic profiles are lacking. There are histologic mimickers, and the pathologic differential diagnosis includes primary tumors from adjacent anatomic sites such as the colorectum, bladder, and gynecologic/Mullerian structures, all of which may extend to and involve the urethra. Hence, IHC is of upmost importance in establishing a diagnosis to guide clinical management, particularly in the cases with initial presentation of metastatic disease.

Immunohistochemistry on the prior reported cases has been reviewed in the English literature in PubMed (Table 1), and no PAX-8 immunoexpression has been documented. The largest, contemporary clinicopathologic study to date of primary mucinous adenocarcinoma of the female urethra was published in 2016 by Harari et al. [10] where they searched through two major academic institutions for cases and found five cases of confirmed primary mucinous adenocarcinoma arising from the female urethra. The mean age of the patients is 67 years, and all presented with at least a pT2 pathologic stage. IHC was performed for GATA3, p63, CK7, CK20, CDX2, ER, PAX-8, and *β*-catenin and showed the following results: positivity for CDX2 in 4/5 (80%) cases, focally positive for CK20 in 4/5 (80%) cases, and focally positive for CK7 in 4/5 cases (80%); p63, GATA3, ER, PAX-8, and *β*-catenin were negative in all cases.

The diagnosis in the current case is particularly challenging because of the immunophenotype, including PAX-8 immunohistochemical positivity. Also, because the tumor showed concurrent CK7 and CK20 immunoexpression, the differential diagnosis included metastatic BRAF-mutated MSI (microsatellite instability) colorectal carcinoma [20]. Extensive imaging, colonoscopy, and pelvic exams were performed to evaluate for a primary malignancy of the bladder, colorectum, and female reproductive system, all of which were ruled out. The patient was ultimately diagnosed with intestinal-type FUA that developed from inflammation-related metaplasia in urethral diverticulum with positive PAX-8 staining. To our knowledge and review of the English literature, this is the first report on PAX-8 immunoexpression. The patient presented with locally advanced disease and pulmonary metastasis, exemplifying the aggressive nature of this particular entity. The patient received chemotherapy and palliative radiation for bone metastasis. Unfortunately, her disease progressed and she was ultimately referred to palliative care. She succumbed to the disease one year after diagnosis.

## 4. Conclusion

FUA is a rare, aggressive tumor, often presenting with metastatic disease. The nonspecific symptomatology often leads to a delay in diagnosis in most patients, and the prognosis is poor in advanced-stage disease. The clinical and pathologic diagnosis can be challenging due to its rarity. Furthermore, delay in presentation has made standardization of treatment difficult to determine, and treatment guidelines currently do not exist. The present report increases the awareness of PAX-8 immunoreactivity in FUA and emphasizes the consideration of this entity in the differential diagnoses in a metastatic tumor, in addition to the importance of clinical and radiological assessment.

## Figures and Tables

**Table 1:** Demographic and immunohistochemical profiles for primary female urethral adenocarcinomas, columnar/mucinous subtype reported in the literature.

Author, year (reference)	Age (years)	Location	IHC	Number of cases (*N*)
Meis et al., 1987 [9]	Mean, 63	U, PU, B, and V	MC+, PAS+	13
Hanai and Lin 1990^~^ [11]	52	U	EMA+, CEA+, AFP+	1
Dodson et al., 1995^∗^ [6]	Unspecified	U	One case PAS+	11
Kato et al., 1998 [12]	71	U and V	CEA+, CA 19-9+, PSA-	1
Murphy et al., 1999 [4]	Unspecified	U	mAbDasl+, PSA-	9
Chan et al., 2000 [13]	72	U	CK7+, CK20+, PSA-	1
Kato et al., 2005^∗∗^ [14]	Mean, 66	U and PU	CEA+	5
Kuroda et al., 2006^∗∗∗^ [15]	77	U, PU, B, and V	CK7+, CA 19-9+, PSA+, CEA+, CA125+, ER-, PAP-, CK20-, PR-	1
Wang et al., 2012 [2]	44 & 52	U, B, and V	Unspecified	2
Hale et al., 2013` [16]	61	U and B	Immunoprofile in detail below`	1
Siosaki et al., 2013 [17]	62	U, B, and V	Unspecified	1
Satyanarayan et al., 2015 [5]	60	U	CK20+, CDX2+, CK7-	1
Harari et al., 2016 [10]	67	U	CDX2+ (80%), focally CK20+ (80%), focally CK7+ (80%), P63-, GATA3-, ER-, PAX-8-, *β*-catenin-	5
Muto et al., 2017“ [7]	69	U	PSAP+, AMACR+, CXD2+, CEA+, MUC2+, CK20+, NKX3.1-, PSA-, ER-, PAX-8-, GATA3-, CK7-, p40-, p63-	1
Gangadhar et al., 2018 [3]	60	U, PU, and V	PAS+	1
Tregnago and Epstein, 2018 [18]	63	PU	CK7+, ER+, focally CDX2+, HMWCK+, NKX3.1-, PSA-, P501S-, AMACR-, p63-, WT1-, PAX-8-, SATB2-, CK20-	1
Qin et al., 2019 [19]	63	U and B	CEA+, CK7+, CK20+, EMA+, p53+	1
Present case	64	U, B, and V	CK7+, CK20+, CDX2+, b-catenin+, CEA+, SATB2+, PAX-8+, TTF-1/Napsin-, p16-, ER-, vimentin-, GATA3-	1

U: urethra; PU: paraurethra; B: bladder; V: vagina; IHC: immunohistochemistry; MC: mucicarmine; PAS: periodic acid–Schiff; EMA: epithelial membrane antigen; CEA: carcinoembryonic antigen; AFP: alpha-fetoprotein; CA 19-9: cancer antigen 19-9; PSA: prostate-specific antigen; CK7: cytokeratin 7; CA125: cancer antigen 125; ER: estrogen receptor; PAP: prostatic acid phosphatase; CK20: cytokeratin 20; PR: progesterone receptor; CDX2: caudal-type homeobox 2; GATA3: GATA binding protein 3; PAX-8: paired box 8; HMWCK: high molecular weight cytokeratin; NKX3-1: NK3 homeobox 1; AMACR: alpha-methylacyl-CoA racemase; WT1: Wilms' tumor 1; SAT-B2: special AT-rich sequence-binding protein 2. ^~^Hanai and Lin report a case with three histologic patterns and partial AFP positivity: intestinal-type cells positive for EMA and CEA, EMA-negative and AFP-positive columnar vacuolated cells, and mainly EMA-positive clear cells. ^∗^Dodson et al. report 11 cases of columnar/mucinous tumors that resembled either endometrial or colonic adenocarcinoma with the exception of one case that resembled prostatic adenocarcinoma. This one case was strongly positive for PSA, and the remaining columnar/mucinous adenocarcinomas were negative for PSA. ^∗∗^One of the five cases had concurrent focal PSA positivity and two of the five cases had chromogranin A positivity. ^∗∗∗^Kuroda et al. report a case with three histologic patterns including columnar/mucinous adenocarcinoma, clear cell adenocarcinoma, and papillary/micropapillary carcinoma without evidence of a cribriform pattern; all three histologic patterns were positive for CK7 and CA 19-9 and focally weak for PSA. Specifically, the columnar/mucinous adenocarcinoma component was also positive for CEA and CA 125. `Hale et al report a case with immunophenotype: PIN-4 (p63, 34*β*e12, and AMACR) positivity for p63 and 34*β*e12 in area of squamous metaplasia and a periurethral glandular remnant and PIN-4 negative in area of glandular metaplasia. An area of residual Skene's gland tissue showed strong nuclear reactivity for p63. The metaplastic glandular cells lacked p63 staining. Nuclear Ki-67 reactivity in areas of squamous metaplasia showed 10% reactivity and in glandular areas was 5%. “Muto et al. reviewed Skene's gland adenocarcinoma, and their literature review revealed six cases of female urethral adenocarcinomas with evidence of a Skene's gland origin; they also reported one original case (shown in the table).

## Data Availability

The data used to support the findings in this case report are included within the article.
